# Oxidative Stress and Heme Oxygenase-1 Regulated Human Mesenchymal Stem Cells Differentiation

**DOI:** 10.1155/2012/890671

**Published:** 2012-02-26

**Authors:** Luca Vanella, Christopher Sanford, Dong Hyun Kim, Nader G. Abraham, Nabil Ebraheim

**Affiliations:** ^1^Department of Biological Chemistry, Medical Chemistry and Molecular Biology, University of Catania, Viale Andrea Doria 6, 95125 Catania, Italy; ^2^Department of Orthopedic Surgery, University of Toledo College of Medicine, Toledo, OH 43614, USA; ^3^Department of Physiology and Pharmacology, University of Toledo College of Medicine, Toledo, OH 43614, USA

## Abstract

This paper describes the effect of increased expression of HO-1 protein and increased levels of HO activity on differentiation of bone-marrow-derived human MSCs. MSCs are multipotent cells that proliferate and differentiate into many different cell types including adipocytes and osteoblasts. HO, the rate-limiting enzyme in heme catabolism, plays an important role during MSCs differentiation. HO catalyzes the stereospecific degradation of heme to biliverdin, with the concurrent release of iron and carbon monoxide. Upregulation of HO-1 expression and increased HO activity are essential for MSC growth and differentiation to the osteoblast lineage consistent with the role of HO-1 in hematopoietic stem cell differentiation. HO-1 participates in the MSC differentiation process shifting the balance of MSC differentiation in favor of the osteoblast lineage by decreasing PPAR**γ** and increasing osteogenic markers such as alkaline phosphatase and BMP-2. In this paper, we define HO-1 as a target molecule in the modulation of adipogenesis and osteogenesis from MSCs and examine the role of the HO system in diabetes, inflammation, osteoporosis, hypertension, and other pathologies, a burgeoning area of research.

## 1. Background

### 1.1. Mesenchymal Stem Cells

 Among cells bone-marrow-derived MSCs have attracted a great deal of attention in the past decade because of their high versatility. MSCs have shown promise in the treatment of cardiovascular disease in a series of animal models. Despite being a rare cell population, MSCs can be extensively expanded *in vitro* thus making them of potential use in the clinic [1]. Human MSCs derived from bone marrow are multipotent cells that differentiate and proliferate into many different cell types in various tissues [1–3]. Bone marrow mononuclear cells can be isolated with ease with a Ficoll-Paque PLUS density gradient. Human MSCs give rise to both osteoblastic and adipogenic lineages when cultured with specific differentiation media. The adipogenic media comprise of complete culture medium supplemented with DMEM-high glucose, FBS, insulin, dexamethasone, and indomethacin. The osteogenic media contain ascorbic acid (for appropriate collagen and extracellular matrix production) and *β*-glycerophosphate (for appropriate mineralization). Several studies demonstrated that an increase in the ROS leads to the elevation of the levels of inflammatory cytokines in adipose tissue. Oxidative stress is a major factor impairing MSCs function resulting in decreased osteogenesis in favor of adipogenesis. Whether MSCs differentiate into osteoblasts or adipocytes is due to multiple signaling pathways including those heavily influenced by HO-1 and -2 [4].

### 1.2. Role of HO during Bone Formation

**Figure 1:**
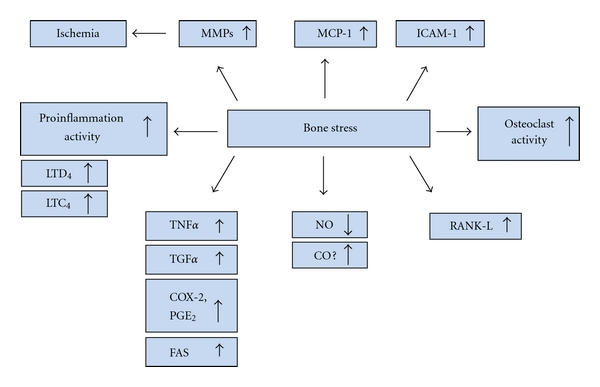
Diagram representing the adverse effects of stress on bone, leading to the releases of inflammatory molecules.

 Recent studies have shown that several growth factors including OGP enhance differentiation of MSCs to osteoblasts [5] and that EGF and OGP signaling pathways enhance osteoblast cell proliferation. Osteogenic growth peptide is a naturally occurring tetradecapeptide that is both an anabolic agent and a hematopoietic stimulator [6]. For example, OGP increases osteoblast proliferation, AP activity, and matrix synthesis and mineralization. It prevents glucocorticoid-induced apoptosis and the subsequent bone remodeling alterations that are associated with steroids [7]. Thus, role of HO in the fluctuations of ROS and its effect on osteonectin levels and in MSC-derived osteoblasts will be described. Cytokines and HO activity have a regulatory role in MSCs microenvironment and hematopoiesis [8]. HO attenuates the overall production of ROS through its ability to degrade the prooxidant, heme, resulting in the production of carbon monoxide, biliverdin/bilirubin, and the release of free iron. These three products of heme degradation play an important role in signaling cascades, cell proliferation and differentiation. HO is the enzyme that catalyzes the rate-limiting step in the degradation of heme and exists in two forms: the inducible HO-1 form and the constitutive HO-2 form [4]. During fracture repair, activation of hypoxia-inducible factor (HIF)-1 and its target genes, VEGF and HO-1, regulate bone remodeling. Bone remodeling is a physiological process which includes bone resorption (by osteoclasts) and bone formation (by the osteoblasts) and requires coordination of three cell types, osteocytes, osteoblasts, and osteoclasts. During aging and in several pathologies, the rate of bone turnover increases, but this is characterized by an impaired osteoblastic bone formation compared to osteoclastic bone resorption caused by decreased number and activity of osteoblastic cells (Figure 1) [9–11].

**Figure 2:**
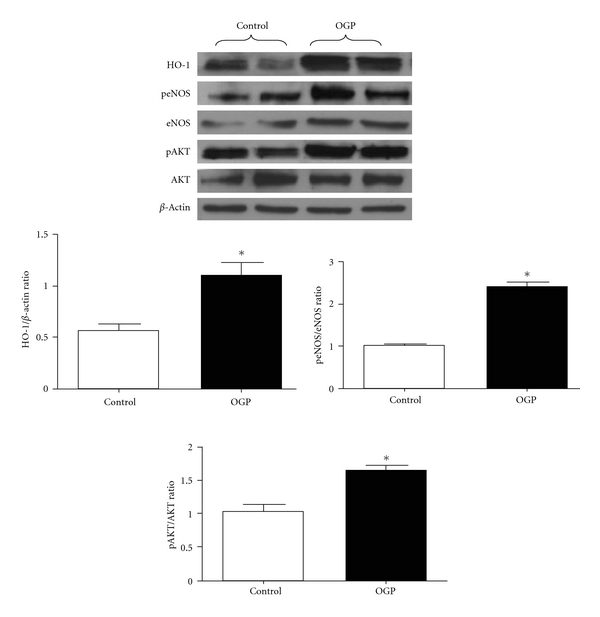
Effect of OGP on HO-1, peNOS/eNOS, and pAKT/AKT proteins expression after 21 days of osteoblast differentiation. Quantitative densitometry evaluation of the proteins ratio was determined. Data are expressed as means ± SEM of three independent experiments; *P < 0.05 control versus OGP.

 This association suggests a role of HO-1 in bone metabolism. As previously stated, HO has strong implications in bone marrow stem cell differentiation [8, 12]. Most notably, HO-1 expression is increased during osteoblast stem cell development. This increase in HO-1 expression precedes an increase in alkaline phosphatase, bone morphogenic protein, osteonectin, and RUNX-2 mRNA [13]. The function of bone-specific alkaline phosphatase has been shown to be that of a biochemical indicator of bone turnover. Upregulation of HO-1 increased MSC-mediated osteoblasts with an associated decrease in adipocytes. OGP increased HO-1 levels [13]. OGP also lead to an increase in pAKT, an antiapoptotic protein, as well as an increase in eNOS and p-eNOS (Figure 2) [13]. Past research has demonstrated that eNOS is an enzyme expressed in osteoblasts that, when deficient, has been shown to lead to a significant reduction in bone formation in murine models [14]. Both eNOS and NO are stimulators of BMP-2 and increase differentiation of osteoblasts [15, 16]. Increased HO-1 expression has also been shown to increase alkaline phosphatase as well as DNA accumulation and mineralization when compared to osteoblasts not treated with OGP [13]. Osteoblasts cultured in hyperglycemic conditions showed reduced levels of bone BMP-2, osteonectin, pAMPK (a signaling molecule in osteoblasts), and eNOS. The reduction of these osteogenic proteins and enzymes was reversed by OGP which upregulated HO-1 with a subsequent increase in BMP-2, HO-1, eNOS, and pAMPK [13]. HO-1 expression and activity are essential for osteoblast differentiation from MSCs. Although basal levels of HO-1 and HO activity are necessary for osteoblast growth, an increase in HO-1 amplifies osteoblast differentiation. Additionally, HO-1 is required to increase pAKT, pAMPK, peNOS levels, and NO bioavailability [17, 18].

## 2. HO-1 and Oxidative Stress

 An additional important association of HO-1 is this enzyme's influence on oxidative stress and reactive oxygen species (ROS). Hyperglycemia and certain cytokines result in an increase in ROS. HO-1 is inhibited in the presence of high glucose. High glucose suppressed HO-1 expression in both cell lines [19–21] and animal models [22–24].

 Intracellularly, reduction-oxidation homeostasis is maintained by the balance between oxidants and antioxidants. Antioxidants including HO, superoxide dismutase, glutathione peroxidase, and catalase are endogenous. Exogenous antioxidants are often derived from food and include vitamins A, C, E, selenium, resveratrol, *α*-tocopherol, and *β* carotene [25–27]. When the natural balance between oxidants and antioxidants is altered, ROS can potentially damage cellular structures like DNA, proteins, and phospholipids. This process, called oxidative stress, is implicated in several neurodegenerative and metabolic diseases including obesity and type 2 diabetes. Moderate-to-severe obesity is associated with an increased risk for hypertension and insulin resistance in humans and animals [22, 28, 29]. The antioxidant effects of HO arise from its ability to degrade heme from destabilized heme proteins and from the production of biliverdin and bilirubin, products of HO with potent antioxidant properties. Heme is a prooxidant so, therefore, its breakdown is antioxidative. Although there appears to be a convincingly clear link between HO-1 and apoptosis, the specific mechanism through which HO-1 prevents apoptosis remains unclear [21, 30, 31]. It is likely that part of the anti-apoptotic role of HO-1 is based on its function as an antioxidant enzyme. However, the possibility remains that one or more of its products play additional roles in anti-apoptotic mechanisms. This association between a reduction in ROS with an increase in HO-1 expression was demonstrated by treating MSCs with CoPP, a strong inducer of HO-1. Exposure of the MSCs to CoPP is effective in decreasing ROS while high glucose concentrations increase ROS. A reduction in ROS permits the restoration of osteoblast markers, specifically induction of osteoprotegerin and osteocalcin [4].

 The discovery that inhibition of HO-1 expression shifts mesenchymal stem cells to favor adipocytic cells at the expense of osteoblastic cells [4, 13] demonstrates that high glucose has an adipogenic potential and also that a direct link is present between HO-1 suppression and an increase in adipogenesis [13]. The exact mechanisms through which HO-1 affects adipocyte and osteoblast differentiation and protects against oxidative injury remain unclear. However, targeting HO-1 expression is a gateway to increasing osteoblast stem cell differentiation, decreasing oxidative stress, and to the attenuation of osteoporosis through the promotion of bone formation.

### 2.1. Introduction of PPAR*γ* and TZD

 There appears to be a strong link between HO-1 and PPAR*γ*. PPAR*γ* has been found to increase with the suppression of HO-1 [13]. The increase in PPAR*γ* is associated with a reduction of HO-1 expression and coupled to a significant increase in adipogenesis [13]. PPAR*γ* is a transcription factor that has been implicated in the development of osteoporosis. PPAR*γ* is considered to be the master regulator of adipogenesis [32, 33], and its expression is believed to increase with age and in diabetics. PPAR*γ* is abundantly expressed in mature adipocytes. Its levels are also increased in the livers of animals that have developed steatosis [34, 35]. Loss of the gene that encodes for PPAR*γ* in embryonic fibroblasts results in the complete absence of adipogenesis in murine studies [36]. In addition, adipocyte differentiation can be stimulated by ectopic expression and activation of PPAR*γ*. Conversely, the absence of this gene results in an increase in osteoblasts and bone mass [37]. PPAR*γ* is expressed in human MSCs [38–40]. This evidence coupled with the fact that bone marrow fat increases and bone marrow osteoblasts decrease with age in both animals and humans [41, 42] demonstrates a possible strong link between PPAR*γ* and osteoporosis. Furthermore, PPAR*γ* has been found to decrease the levels of core-binding factor alpha (Cbfa1) and RUNX-2, two factors needed for osteoblast development. By leading to the differentiation of adipocytes over osteoblasts, activation of PPAR*γ* subsequently leads to a decrease in bone mineral density and appears to be strongly correlated to the development and progression of osteoporosis. The stimulation of PPAR*γ* occurs following upregulation of PPAR*γ* expression by TZD [43]. TZDs provide diabetics with many benefits including lower blood glucose levels and reduced rates of atherosclerosis. However, these benefits come with multiple negative effects on bone such as inhibiting osteoblast formation [44–46] and inducing apoptosis of mature osteoblasts. Activation of PPAR*γ* by TZDs also results in the suppression of many vital osteogenic transcription factors in both animal models and humans [47, 48]. The end result of these antiosteogenic effects is a decrease in bone mass. Furthermore, TZD use has been shown to increase the risk of fracture in postmenopausal women [49–51]. A large cohort study showed that the use of TZDs in type 2 diabetics increased the fracture risk in women over 65 years old. This increased fracture risk was seen after only one year of TZD use [51]. The association between TZD and fracture risk is likely due to a discrepancy between bone formation and resorption as rosiglitazone, a thiazolidinedione, has been found to decrease bone mass in the hip and decrease serum osteocalcin levels [52]. Aging leads to a decrease in HO-1 and an increase in bone marrow adiposity with bone loss and an increase in the expression of PPAR*γ* and, ultimately, PPAR*γ* [53]. Extensive research demonstrates that PPAR*γ* plays a pivotal role in regulating MSC specification towards adipogenesis versus osteogenesis and is instrumental in governing both bone mineral density and osteoporosis.

### 2.2. HO-1 and CYP-450

 HO-1 induction increased the levels of cytochrome P450- (P450-) derived epoxyeicosatrienoic acids (EETs), which further decreased PPAR*γ* but increased osteoblasts. We examined if EET levels regulate the MSC-derived adipocytes. Expression of FAS and PPAR*γ* levels was significantly (*P* < 0.05) increased in preadipocyte (14 days of MSC-derived adipocyte differentiation), and conversely pACC and *β*-catenin were decreased (*P* < 0.05) in preadipocyte. The increase in FAS and PPAR*γ* in preadipocyte was suppressed by the EET. In contrast, the EET treatments significantly increased both pACC and *β*-catenin compared to cell treated with vehicle solutions. Further, MSCs-derived adipocyte exhibited a significantly higher expression of PPAR*γ*, SREBP-1, and GLUT4 compared to MSCs-adipocyte grown in the presence of EET [54]. Adipocyte cultured in the absence of the EET agonist showed HO-1 levels that were decreased at day 10 and day 15 compared to adipocyte cultured in the presence of EET. In contrast PPAR*γ* and C/EBP*α* protein pattern was the reverse by inhibition of HO-1. PPAR*γ* and C/EBP*α* levels were significantly increased, while Wnt/*β*-catenin protein levels were decreased when compared with adipocytes cultured in the presence of EET. EET-agonist-treated cells showed an increase in *β*-catenin. HO-1 expression was greatly diminished during adipogenic differentiation of MSCs, while FAS and PPAR*γ* levels increased. Ectopic expression and activation of PPAR*γ* are sufficient to induce adipocyte differentiation [33]. FAS mRNA levels increased dramatically during 3T3-L1 adipocyte differentiation [55]. In agreement with previous studies, Wnt stimulation facilitates disruption of the axin-based complex [56, 57]. This results in a decrease in the phosphorylation of *β*-catenin, which enhances *β*-catenin accumulation and activation leading to an arrest in adipogenesis at the early progenitor stage through the blocking of PPAR*γ* signaling [58, 59]. Furthermore, EETs inhibited MSC-derived stem cell adipogenesis presumably through activation of HO-1/Wnt/*β*-catenin and the expression of C/EBP*α*, a marker of adipocyte differentiation [54]. In the present study, the increase of C/EBP*α* was prevented by treatment of an EET agonist at day 10 and day 14. Vanella and coworkers provided direct evidence that EET-agonist-induced activation of HO-1 led to the increase in adiponectin and phosphorylation/inactivation of ACC and consequently decreased of FAS levels.

### 2.3. Diabetes and HO-1

 Diabetes directly affects the integrity and functionality of bone in both humans and animals resulting in osteoporosis and an increase in adipogenesis [60–64]. Patients with diabetes frequently have a lower bone mineral density with associated osteopenia or osteoporosis. Because diabetic individuals commonly have decreased bone mass and bone mineral density, they are more susceptible to fractures and impaired bone healing. This correlation between diabetics and an increase in fractures has been well documented in the literature. A large prospective cohort study of over 32,000 postmenopausal women found that those with type 1 diabetes mellitus were 12 times more likely to experience hip fractures than those women without type 1 diabetes. Women with type 2 diabetes had a 1.7-fold increase in hip fractures when compared to women without this disease [65]. Prevention at decreasing osteoporotic fractures in patients with diabetes can involve strict glucose control, prevention and treatment of vascular complications, regular exercise, and fall prevention [66]. Additionally, biochemical markers of bone turnover are negatively affected in diabetics [67]. One such biomechanical marker, osteocalcin, has been found to be decreased in patients with diabetes [68]. However, the exact pathogenesis of the reduction in bone mass seen in diabetics is unknown. Also, the effect of osteoblast stem cell differentiation under high glucose conditions has not been fully elucidated although, as previously stated, strong evidence supports the belief that hyperglycemic environments decrease osteoblast differentiation and increase adipocyte differentiation [4, 13]. Upregulation of HO-1 expression in obesity and type 2 diabetes results in a decrease in visceral and subcutaneous fat content, improved insulin sensitivity, and increased insulin receptor phosphorylation [22, 69–71]. MRI studies showed that upregulation of HO-1 decreased adiposity and adipocyte hypertrophy [17, 70]. The decrease in HO-1 expression was associated with an impairment in the mesenchymal stem cells production of adiponectin and increased adipogenesis [13, 22]. In addition, HO-1 gene expression has a differential effect on osteoblasts and adipocyte cell proliferation and differentiation [4, 13]. EETs administration decreased adiposity and insulin resistance in mice and rat models of obesity and diabetes via an increase in HO-1 gene expression and signaling cascade including the activation of AMPK and pAKT [72–74]. Sacerdoti and coworkers [75, 76] studied the interactions between HO-1 gene expression and EETs in vitro and showed that EETs induce HO-1 protein and HO activity. Human stromal-mesenchymal stem cells express CYP450 monooxygenase and form EETs and 20-HETE [8]. Not only 20-HETE but other eicosanoids are correlated with the progression of diabetes [77, 78] as well.

 MSCs have the ability to metabolize arachidonic acid to HETE at comparable levels to endothelial cells. Additionally, the EET agonist (NUDSA) inhibited soluble epoxide hydrolase (sEH) and reduced the rate of body weight gain in obese mice which was accompanied by an increase in HO-1 expression [73, 79]. EET crosstalks with HO-1 on the decrease of adipogenesis [54]. The ability of hyperglycemic conditions and diabetes to activate adipocytes to undergo adipogenic differentiation appears to be strongly influenced on the suppression of HO-1. Therefore, it seems that osteoblast differentiation under hyperglycemic conditions decreases secondary to this suppression of HO-1. In addition, high levels of glucose lead to an increase in reactive oxygen species which have been shown to block BMP-2, osteonectin, osteoprotegerin and osteocalcin [4]. A 2010 study used osteoblast-like MG63 cells to demonstrate that hyperglycemic conditions significantly suppressed cell growth, mineralization, and expression of multiple osteoblastic markers (RUNX-2, osteocalcin, osteonectin, type 1 collagen). These high glucose conditions simultaneously stimulated the expression of PPAR*γ*, adipocyte fatty-acid-binding protein (aP2), resistin, and adipsin, all markers of adipogenesis [4].

**Figure 3:**
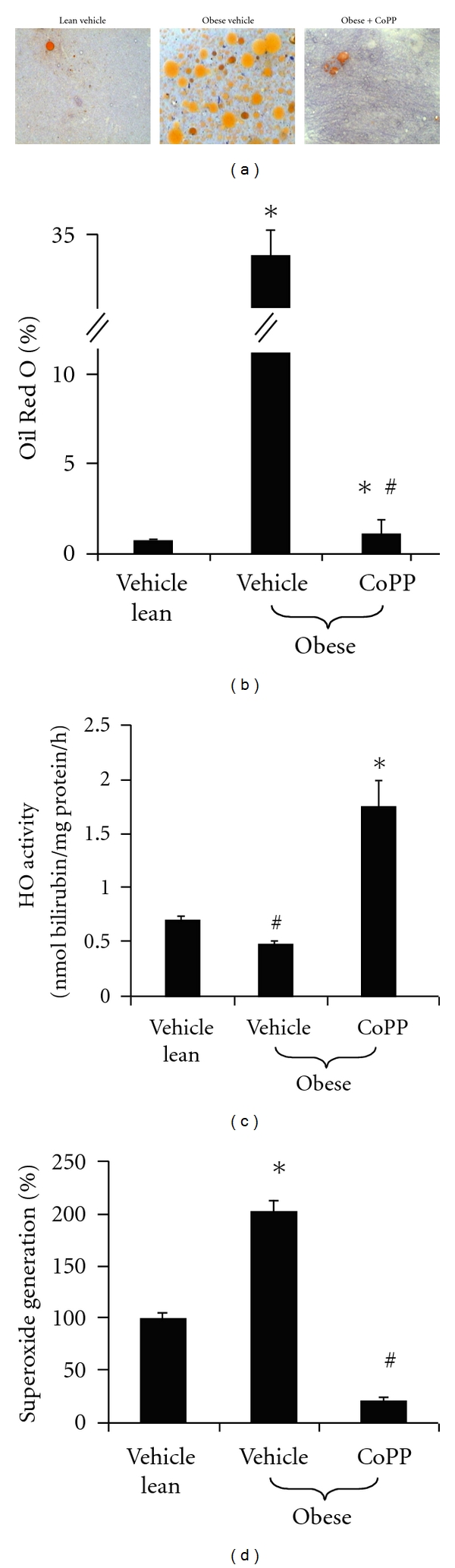
Evaluation of lipid content measured as percentage of Oil Red O staining. (a) Representative photographs demonstrating an increase in lipid droplets in the bone marrow of obese mice compared to lean mice and obese mice treated with CoPP. (b) Quantitative analysis of bone marrow lipid content showing a significant increase in Oil Red O staining in the obese vehicle. Please note bars on the graphs represent the mean ± SEM of three independent experiments; *P < 0.05 versus vehicle-treated lean mice; ^#^ *P* < 0.05 versus vehicle-treated ob mice. (c) HO activity of bone marrow cells showing an increase in HO activity in both lean mice and obese mice treated with CoPP when compared to obese mice not receiving treatment. Please note bars on the graphs represent the mean ± SEM of four independent experiments; ^#^ *P* < 0.05 versus vehicle-treated lean mice; *P < 0.01 versus vehicle-treated ob mice. (d) Superoxide generation in bone marrow cells is shown to be increased in obese mice and is lowest in the mice treated with CoPP implying an antioxidant effect of HO. Please note bars on the graphs represent the mean ± SEM of four independent experiments; *P < 0.05 obese versus vehicle lean; ^#^ *P* < 0.001 versus vehicle-treated obese.

**Figure 4:**
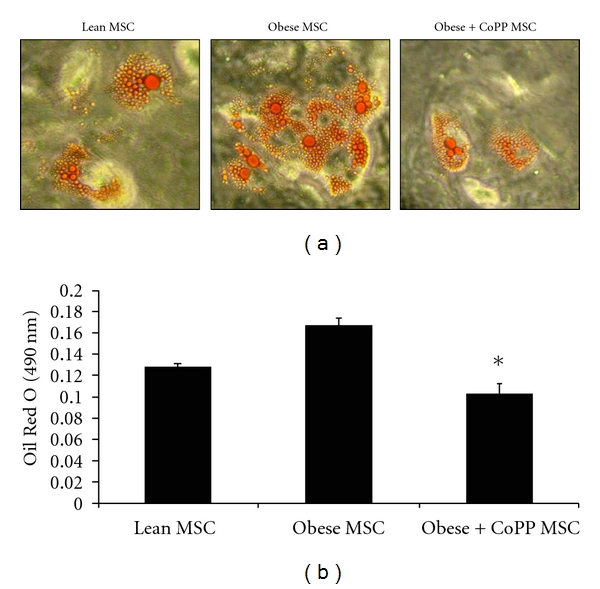
Effect of CoPP on MSC-derived adipogenesis. Adipogenesis was measured as the relative absorbance of Oil Red O at day 10. (a) Representative pictures demonstrating an increase in lipid droplets in the MSC of obese mice compared to lean mice and obese mice treated with CoPP. (b) Quantitative analysis of adipogenesis showing a significant increase in Oil Red O staining in the obese MSC-derived adipocytes. Data are expressed as mean ± SE (*P < 0.01 obese versus CoPP). Note: unpublished data.

 An additional unpublished study by the authors provides evidence that obese mice have a significant increase in bone marrow adipocytes when compared to both lean mice and obese mice treated with the potent HO-1 inducer CoPP (Figures 3(a), 3(b), and 4). The HO activity in the bone marrow was significantly lower in obese mice compared to lean mice. However, CoPP treatment did increase the HO activity in obese mice (Figure 3(c)). Lastly, superoxide levels were increased in obese mice compared to lean mice. CoPP treatment to obese mice decreased these elevated superoxide levels (Figure 3(d)).

 Furthermore, hyperglycemia has been shown to promote cell death in many cell types such as endothelial and mesangial cells [19, 80, 81]. Oxidative stress is a consequence of hyperglycemia that can induce apoptosis via signaling and/or outright molecular damage. In streptozotocin- (STZ-) induced diabetic rats, symptoms of oxidative stress, including increases in cellular heme and apoptosis, were found to be reversed by upregulation of HO-1 [23]. Elevated glucose levels cause glucose oxidation which leads to an increase of ROS in endothelial cells. This formation of ROS is believed to be the major factor in endothelial dysfunction such as abnormalities in cell cycling and delayed replication. However, these abnormalities are able to be reversed by antioxidant agents and an increased expression of antioxidant enzymes. Overexpression of HO-1 has been shown to make cells resistant to oxidative stress-causing agents while enhancing cell growth and angiogenesis. Such findings further demonstrate the important cytoprotective and antioxidant role of HO-1. Strong evidence exists that diabetic-induced hyperglycemia has a substantial impact on the development and progression of osteoporosis. The stimulation of osteoblast apoptosis as well as the decrease in osteoblast and increase in adipocyte differentiation all contribute to the decreased bone mineral density observed in individuals with diabetes.

### 2.4. Future Perspectives

**Figure 5:**
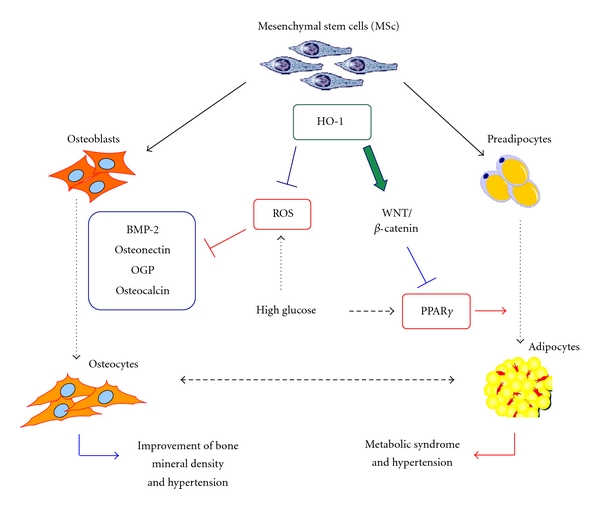
Schematic demonstrating the interplay between HO-1, hyperglycemia, ROS, and PPAR-*γ* on the regulation of osteoblast and adipocyte differentiation from mesenchymal stem cells.

 Osteoporosis is a complex condition with significant morbidity, characterized by low bone mass, increased fragility, and fracture risk. Although mechanisms are unclear, there is significant evidence showing the interplay between HO-1, PPAR*γ*, oxidative stress, and hyperglycemia. All have a role in the regulation of mesenchymal stem cell differentiation and the development of osteoporosis (Figure 5). Several studies have shown that HO-1 plays a considerable role in reducing oxidative stress, cellular apoptosis, increasing osteoblast differentiation, while simultaneously suppressing adipocyte differentiation from mesenchymal stem cells. HO-1 upregulation causes an increase in alkaline phosphatase and BMP-2 expression and a decrease in PPAR*γ* expression, increasing bone formation and decreasing adipocyte differentiation. Conversely, HO-1 suppression upregulates PPAR*γ* and increases adipogenesis, worse in diabetics, with reduced bone mineral density. Pharmacological and genetic approaches to deliver HO-1 or products of heme degradation remain to be elucidated for the causes, treatment, and prevention of osteoporosis.

## Abbreviations

HO-1: Heme oxygenase-1
MSCs: Mesenchymal stem cells
PPAR*γ*: Peroxisome proliferator-activated receptor gamma
GSK3*β*: Glycogen synthase kinase 3*β*
aP2: Fatty-acid-binding protein 4 (FABP4)
EGF: Endothelial growth factor
Wnts: Wingless-type
C/EBP*α*: Adipogenic transcription factors CCAAT/enhancer binding protein *α*
ROS: Reactive oxygen species
NO: Nitric oxide
BMP-2: Bone morphogenic protein-2
RUNX-2: Runt-related transcription factor 2
OGP: Osteogenic growth peptide
P450: Cytochrome P450
EET: Epoxyeicosatrienoic acids
TZD: Thiazolidinediones
CoPP: Co-protoporphyrin IX
SnMP: Tin-mesoporphyrin IX
AP: Alkaline phosphatase
VEGF: Vascular endothelial growth factor.
